# Detecting differential allelic expression using high-resolution melting curve analysis: application to the breast cancer susceptibility gene CHEK2

**DOI:** 10.1186/1755-8794-4-39

**Published:** 2011-05-11

**Authors:** Tú Nguyen-Dumont, Lars P Jordheim, Jocelyne Michelon, Nathalie Forey, Sandrine McKay-Chopin, Olga Sinilnikova, Florence Le Calvez-Kelm, Melissa C Southey, Sean V Tavtigian, Fabienne Lesueur

**Affiliations:** 1 Genetic Cancer Susceptibility Group, IARC, 69372 Lyon, France; 2 INSERM U590, Université Lyon 1, Lyon, France; 3 Peter MacCallum Cancer Center, East Melbourne 2, VIC 3002, Australia; 4 Unité Mixte de Génétique Constitutionnelle des Cancers Fréquents, Hospices Civils de Lyon, Centre Léon Bérard, 69373 Lyon, France; 5 Department of Pathology, The University of Melbourne, VIC 3010, Australia; 6 Department of Oncological Sciences, Huntsman Cancer Institute, University of Utah, Salt Lake City, UT 84112, USA

## Abstract

**Background:**

The gene *CHEK2 *encodes a checkpoint kinase playing a key role in the DNA damage pathway. Though *CHEK2 *has been identified as an intermediate breast cancer susceptibility gene, only a small proportion of high-risk families have been explained by genetic variants located in its coding region. Alteration in gene expression regulation provides a potential mechanism for generating disease susceptibility. The detection of differential allelic expression (DAE) represents a sensitive assay to direct the search for a functional sequence variant within the transcriptional regulatory elements of a candidate gene. We aimed to assess whether *CHEK2 *was subject to DAE in lymphoblastoid cell lines (LCLs) from high-risk breast cancer patients for whom no mutation in *BRCA1* or *BRCA2* had been identified.

**Methods:**

We implemented an assay based on high-resolution melting (HRM) curve analysis and developed an analysis tool for DAE assessment.

**Results:**

We observed allelic expression imbalance in 4 of the 41 LCLs examined. All four were carriers of the truncating mutation 1100delC. We confirmed previous findings that this mutation induces non-sense mediated mRNA decay. In our series, we ruled out the possibility of a functional sequence variant located in the promoter region or in a regulatory element of *CHEK2 *that would lead to DAE in the transcriptional regulatory milieu of freely proliferating LCLs.

**Conclusions:**

Our results support that HRM is a sensitive and accurate method for DAE assessment. This approach would be of great interest for high-throughput mutation screening projects aiming to identify genes carrying functional regulatory polymorphisms.

## Background

The *CHEK2 *gene (cell cycle checkpoint kinase 2) is a multiorgan tumour susceptibility gene involved in the maintenance of genomic stability. CHEK2 functions downstream of ATM (Ataxia-telangiectasia mutated) to phosphorylate several substrates, including TP53 (Tumour protein p53), Cdc25C (Cell division cycle 25C) and BRCA1 (Breast cancer 1, early onset), leading to cell cycle arrest, activation of DNA repair or apoptosis in response to DNA double-stranded breaks. Since *CHEK2 *plays a key role in the DNA damage pathway, loss of function of the protein may allow cells to evade normal cell cycle checkpoints, ultimately leading to tumour initiation or progression. The CHEK2*1100delC deletion, falling in the kinase domain of the protein, has been widely studied for its contribution to inherited breast cancer susceptibility [1]. This mutation induces a premature stop codon in exon 10, and causes the truncation of the protein at codon 381 thus abrogating its kinase activity. The frequency of CHEK2*1100delC differs between ethnic populations, and is higher in the North of Europe and low or absent in other countries [2].

The CHEK2-Breast Cancer Consortium reported a frequency of 5.1% for the CHEK2*1100delC variant in familial breast cancer cases who tested negative for *BRCA1 *and *BRCA2 *(Breast cancer 2, early onset) mutations, as opposed to 1.1% of carriers in the control population [3]. This intermediate-risk breast cancer susceptibility allele almost triples the risk of developing the disease in unselected breast cancer cases (OR = 2.34; 95% CI[1.72 - 3.20]) [4]. Other founder mutations in *CHEK2 *have been associated with an increased risk of cancer [5]. Though first discovered in breast cancer patients, *CHEK2 *mutations have since been reported to predispose to a range of cancer types, including ovarian, prostate, kidney and colorectal cancers [6], supporting the hypothesis that *CHEK2 *is a multiorgan cancer susceptibility gene [5].

As part of an international breast cancer genetics study aiming to investigate candidate genes conferring an intermediate-risk of breast cancer, we mutation screened the coding exons and the adjacent proximal introns of *CHEK2 *in 1415 cases and 1204 controls. The main goal of this study was to evaluate and to compare the role of truncating mutations, splice junction mutations and rare missense substitutions in breast cancer susceptibility [7]. In order to fully assess the contribution of *CHEK2 *in breast cancer susceptibility, we aimed to test whether the gene was subject to differential allelic expression (DAE). In such a case, it would be worth extending variant discovery efforts from the coding sequence of the gene to known or predicted regulatory regions to search for causal variants. Indeed, phenotypic variation may be influenced by sequence variations in genes by alterations in the quality or in the quantity of the encoded proteins [8]. These changes are transmitted from the gene to the protein in the guise of modifications of the sequence or the abundance of mRNA. From this perspective, it can be hypothesized that gene expression regulation may be the underlying explanation for a proportion of cancer that have not been resolved yet by mutation screening of coding regions in currently known cancer predisposition genes.

Allelic imbalance was first described in parental imprinting and X-chromosome inactivation but it is becoming clear that *cis*-acting variations in gene expression occur commonly in the human genome, playing a key-role in human phenotypic variability [9-11]. Characterization of the effect of *cis*-acting polymorphisms in regulatory regions is a great challenge due to the difficulty to locate these regions. In addition, regulatory variants are not robustly detected by sequence analysis since SNP identification by screening regulatory regions does not consistently allow prediction of the effect of observed SNPs on gene expression. Thus, knowledge of the effect of genetic variants affecting mRNA transcription is very limited. One possible approach to address this issue is the examination of disruption/alteration of gene expression level. The most sensitive test for this phenomenon is to carry a careful survey of whether two alleles of a gene are equally expressed. This approach has been used in studies aiming at identifying functional cis-variants that can have a role in susceptibility to breast [12,13] and colorectal cancer [14,15]. In some cases, observation of DAE will be explained by a truncating mutation resulting in non-sense mediated mRNA decay (NMD) or by a splice junction mutation resulting in an unstable transcript. However, DAE can also be the signature of a heterozygous carriage of a regulatory variant [16] or of an epigenetic event (methylation) [17].

In this study, we used a high-resolution melting (HRM) analysis approach to perform allele-specific expression measurement in *CHEK2*. As in currently used methods for investigating DAE, this approach is applied to individual subjects who are heterozygous for an exonic marker SNP, specifically targeted by a labelled probe, called SimpleProbe [18,19]. Data acquisition on HRM instruments consists of monitoring changes in the fluorescence intensity of the probe, as it dissociates from the two allelic templates, while the probe-target duplexes are continuously heated. We have already reported the use of this methodology to compare the relative abundance of allelic transcripts in a study investigating mRNA degradation due to NMD in *BRCA2 *[19], and in a group of selected genes involved in the cellular response to the cytotoxic agent cytarabine [20]. In these studies, DAE analysis was limited by the single-capillary throughput of the HRM device used, the HR-1™ instrument, and allelic imbalance was quantified manually. Here, we report additional experiments and testing, as well as up-scaling possibilities with a high-throughput HRM device, the LightScanner^® ^instrument that uses a 384-well plate format. To improve the analysis of allelic expression, an analysis tool was developed using R in order to process data acquired with HRM commercialized software. Our script provides allelic imbalance estimates and subsequent statistical calculations that are required to assess DAE.

## Methods

### Lymphoblastoid cell lines

We used a total of 89 lymphoblastoid cell lines (LCLs) derived from breast cancer patients, who were considered to be at high risk of carrying a genetic predisposition to cancer due to an early age at onset and/or family history, and for whom no mutation in *BRCA1 *or *BRCA2 *genes had been identified. Biological samples were obtained from Creighton University School of Medicine (Omaha, NE, USA, 33 familial cases), Centre Léon Bérard (CLB, Lyon, France, 21 patients diagnosed below age 50) and the Kathleen Cuningham Consortium for Research into Familial Aspects of Breast Cancer (kConFab, Melbourne, Australia, 35 familial cases). LCLs were established by Epstein-Barr virus immortalization of patients' blood lymphocytes. Cells were maintained in RPMI 1640 medium (Invitrogen, Cergy-Pontoise, France) supplemented with 20% fetal calf serum (VWR, Fontenay-sous-bois, France), 0.4% fungizon (Qiagen, Courtaboeuf, France) and 1% penicilin-streptomycin (Invitrogen), in 5% CO2 incubator at 37°C with 95% humidity. For NMD inhibition, LCLs were treated for 6 hours with 100 *μ*M puromycin (Sigma Aldrich, St Quentin Fallavier, France).

### DNA samples

Genomic DNAs and total RNAs were extracted from LCLs using Puregene DNA isolation kit (Qiagen) and NucleoSpin RNA II kit (Machery Nagel, Hoerdt, France), respectively. Integrity of RNA was controlled using the BioAnalyzer and RNA NanoChip II kit (Agilent, Massy, France) according to the manufacturer's instructions. RNAs harbouring an RNA integrity number (RIN) ≥ 8 were selected for further analysis [21]. Whenever the quality threshold was not reached, a new RNA extraction was performed so that all the RNAs used in this study had a minimum RIN of 8. Complementary DNA (cDNA) synthesis was performed from 1 *μ*g total RNA using SuperScript™ III First Strand Synthesis System for RT-PCR (Invitrogen) with oligo(dT) primers, according to the manufacturer's instructions.

### Mutation screening

The 89 subjects included in this study were drawn from a large-scale case-control mutation screening study involving 1415 cases and 1204 controls, that has been described elsewhere [22,23]. CHEK2*1100delC carriers were all confirmed by direct sequencing on genomic DNA (For mutation screening results, see Additional file 1).

### PCR amplification for DAE assessment

DAE was assessed in four replicates of primary PCR (PCR_1_), both with cDNA, cDNA from puromycin-treated LCL, and genomic DNA (For primers and probes, see Additional file 2). PCR_1 _contained 2 *μ*l template DNA in 1× PCR Buffer, 1.5 mM MgCl2, 0.13 mM dNTP, 0.2 *μ*M forward and reverse primers specific to genomic DNA or cDNA, and 0.05 Units Platinum Taq Polymerase (Invitrogen), in a final volume of 8 *μ*l. The temperature cycling protocol was: 94°C for 3 minutes; 30 cycles at 94°C for 30 seconds, 62°C for 45 seconds and 72°C for 30 seconds; and finally 72°C for 5 minutes. To reduce competitive binding of the probe and the complementary strand during the melting curve analysis, the secondary PCR (PCR2) was carried out asymmetrically, with the primer generating the target strand at a 5-fold higher concentration (0.5 *μ*M) than the primer for the other strand (0.1 *μ*M). In addition, PCR_2 _contained 2 *μ*l of 1:15 diluted in TE^-4 ^PCR1 products combined with 0.9× Buffer, 1.38 mM MgCl2, 0.12 mM each dNTP, 0.5 *μ*M SimpleProbe (Tib Molbiol, Berlin, Germany) and 0.4 Units of Taq Platinum Polymerase in a final volume of 6 *μ*l. Clear oil (Avatech) saturated with Tween 80 (Sigma Aldrich) was used to overlay PCR reactions. The temperature cycling protocol was the same as above, except that 45 cycles were performed. DAE analyses were performed in batches of 96 samples, corresponding to 4 replicates of genomic DNA, 4 replicates of cDNA and 4 replicates of puromycin-treated cDNA derived from 8 different LCLs.

### High-resolution melting analysis

PCR product melting curves were obtained from the HR-1™ and the LightScanner^® ^instruments by melting from 35°C to 75°C. Data were obtained with the supplied software (HR-1™ v1.5 and LightScanner^® ^Software v2.0, respectively), and then exported to an analysis tool that we developed in R, a programming language and software environment for statistical computing and graphics http://cran.r-project.org. R scripts were developed in order to retrieve the data, to apply the Savitsky-Golay filter to smooth the derivative melting curves and to calculate the peak heights. For each sample, ratios were measured from 4 PCR-replicates and the mean ratio was calculated across all replicate samples. The R scripts are available on http://sourceforge.net/projects/hrmdae. The level of allelic imbalance for each individual was determined from the difference between the log of the signal ratio in cDNA and the corresponding log ratio in genomic DNA. Statistical significance for the allelic imbalance was calculated using Student's t-test. Criteria for DAE were the following: i) the point estimate of the difference between genomic DNA and cDNA ratios should be greater than 20%; ii) the Student's t-test p-value should be ≤ 0.05, and iii) the 95% confidence interval of the point estimate should not include 0 [14,19,20,24].

## Results

### Genotyping of CHEK2 exonic SNPs

The main goal of the initial case-control mutation-screening project was to identify rare, potentially pathogenic genetic variants within the coding sequence and the proximal intronic splice consensus sequences of *CHEK2*. This mutation screening simultaneously provided the genotype of all common coding SNPs for every subject enrolled in the molecular epidemiology study. For 89 of the breast cancer patients investigated, LCLs were available to conduct DAE analysis.

In order to make a differential measurement of the level of expression of the two alleles of a gene for a given patient, one must be able to distinguish between the alleles. We used the two most common exonic SNPs that were identified during the mutation screening process, namely rs2236142 and rs2236141, and only the cell lines that are heterozygote for at least one of the two SNPs were selected for further analysis. These two markers are reported to be common in the dbSNP database (Minor allele frequency of 49.2% and 25.4% in European populations, respectively). Thirty-two out of 89 cell lines were heterozygotes for rs2236142 and 17 out of 89 were heterozygotes for rs2236141 (see Additional Table 1). Eight individuals were double heterozygotes.

**Table 1:** Comparison of the duration of the DAE analysis between the HR-1™ and the LightScanner^® ^instruments, for 96 samples

DAE step	HR-1™ instrument	LightScanner^® ^instrument
PCRs	Same duration	Same duration

Data acquisition	2 days	12 minutes

Data analysis	1 full day	15 minutes

The HR-1™ instrument can only analyze a single sample per run making data analysis time consuming. The LightScanner^® ^instrument, with its 384-well plate format, is of greater practical efficiency. Data analysis was performed using an analysis tool that we developed.

### Evaluation of the HRM method to detect DAE

**Figure 1:**
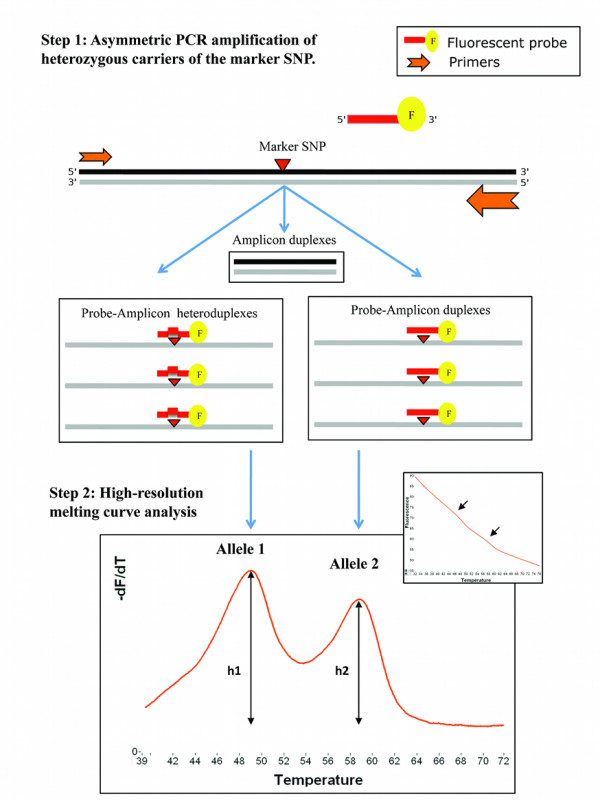
**Principle of high-resolution melting curve analysis (HRM) for detection of allelic expression imbalance**. A single labelled fluorescent probe is designed with complete complementarity to one allele of the exonic SNP chosen as marker, while mismatching the other allele. Following an asymmetric PCR reaction in presence of the probe, HRM analysis allows the alleles in heterozygous individuals to be distinguished by differences in their melting temperatures (Tm), with a fluorescent signal correlated to the relative abundance of each transcript. The Allele 2/Allele 1 ratio is calculated as h2/h1.

This technique relies on the distinction between the two alleles in heterozygous individuals using differences in melting temperature (Tm) with a derivative fluorescent signal correlated to the relative abundance of each transcript. We first verified that HRM could distinguish between the two alleles of each SNP in our experimental conditions, by assaying genomic DNA and cDNA from all three genotypes. Analysis of the melting curves of the homozygous samples showed a transition at a Tm specific to each allele (Figure 1). Melting transitions were converted into peaks on the derivative plot. Heterozygous samples presented transitions and peaks corresponding to each allele at both Tm. A no-template control was taken as baseline to subtract local background value to the fluorescence intensity of the samples.

To examine the feasibility of detecting DAE by melting curve analysis, we created a range of melting curves associated with known allelic imbalance. Using homozygous genomic DNAs, we produced bi-allelic templates with increasing minor allele:major allele proportions (9:1, 8:2, 7:3, 6:4, 5:5, 4:6, 3:7, 2:8, 1:9).

**Figure 2:**
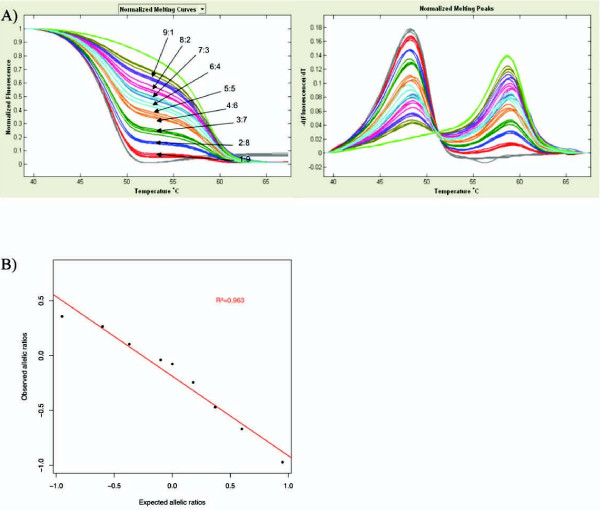
**Mixing experiment to assess efficiency of HRM for detection of differential allelic expression**. (A) SimpleProbe^® ^melting curves generated on the LightScanner^® ^instrument from mixing series of opposite homozygous genomic DNAs for the marker SNP rs2236142 in CHEK2. Mixing ratios are indicated on the figure (G allele: C allele ratio). (B) The determination coefficient (R2) between the expected and the observed allelic ratios was 0.963. Each value corresponds to the mean value of 4 replicate measurements.

Allelic (im)balance was observed as the ratio of the peak heights of the fluorescence signal. As expected, the melting profiles of the mixtures of opposite homozygotes reflected the relative contribution of each allele to the total mixture (Figure 2A). For both SNPs we observed good correlations between allelic imbalance and peak height ratio measurements. For rs2236142, R^2 ^= 0.974 on HR-1™ and R^2 ^= 0.963 on LightScanner^® ^(Figure 2B); for rs2236141, R^2 ^= 0.973 on HR-1™ and R^2 ^= 0.963 on LightScanner^® ^(data not shown). The mixing experiments showed that the measured allelic ratios varied in a linear relationship with the dilution ratios. Altogether, these results show that HRM is able to accurately detect different extents of DAE.

### Assessment of DAE for CHEK2 in LCLs from breast cancer patients

Mutation screening of the 89 LCLs identified four carriers of the CHEK2*1100delC mutation (see Additional file 1). This mutation induces a premature termination codon and has been reported to trigger the NMD pathway [25,26], which leads to the specific degradation of mRNAs bearing such deleterious mutation [27]. In order to distinguish DAE that would be caused by NMD from DAE that would be caused by a regulatory variant altering the level of expression of the transcript, cDNA was derived from LCLs treated and untreated with puromycin, from each individual.

We performed quantitative measurements on genomic DNA and on both types of cDNA. Genomic DNA served as an internal control and provided the expected peak heights ratio value for a 1:1 allelic ratio, thereby controlling for any bias in the binding of the fluorescent probe to the two alleles. Because of differences in fluorescence yield, measured peak heights ratios differed from unity when genomic DNAs were assessed. However, the melting profiles of genomic DNA were in accordance with what was expected from the mixing experiment.

**Figure 3:**
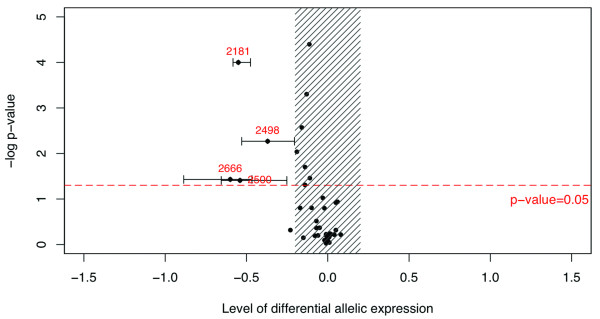
**R plot showing the DAE assay results for the 41 heterozygous individuals enrolled in the study**. The level of DAE is calculated by dividing the allelic ratio in cDNA by the corresponding ratio in genomic DNA (log cDNA-log gDNA). Statistical significance for DAE is evaluated using Student's t-test. Evidence for DAE is reached when i) the point estimate of the level of DAE (plotted on the horizontal axis) is greater than 20%, ii) the Student's t-test p-value (plotted on the vertical axis) is ≤ 0.05, and iii) the 95% confidence interval of the point estimate (based on 4 replicate assays) does not include 0. Samples above the horizontal line and outside the hatched area reached the statistical threshold for DAE. In our experiment, four samples met all criteria (Samples 2181, 2498, 2500 and 2666).

**Figure 4:**
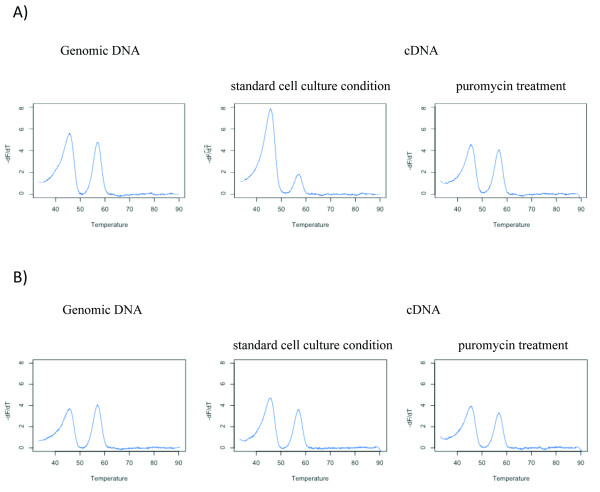
Non-sense mediated mRNA decay causes differential allelic expression in CHEK2*1100delC carriers. Allelic ratio measurements were performed on genomic DNA (gDNA), cDNA derived from LCLs in standard cell culture condition, and cDNA from LCLs treated with puromycin, an NMD inhibiting agent. (A) For a carrier of the mutation, comparison of gDNA and cDNA melting profiles supports the existence of DAE. Puromycin-cDNA profile resembles gDNA, supporting the role of NMD in the DAE observed in this individual. (B) The wild-type sample shows similar profiles in all three situations. HRM profiles were generated with the R script.

The first series of analysis using the SNP rs2236142 as marker included 32 heterozygous individuals for this coding SNP. The statistical threshold for DAE was reached in four individuals (Figure 3). Mutation screening results indicated that these four patients carried the CHEK2*1100delC mutation (see Additional file 1). Observed levels of DAE varied from 37% to 60%, revealing a substantial expression imbalance of an order likely to have biological importance. NMD inhibitory treatment on these four LCLs showed melting curves profiles tending towards the genomic curve profile, which is the reference for a 1:1 allelic ratio (Figure 4A). This confirms previous findings that the CHEK2*1100delC mutation leads to allele-specific degradation by triggering the NMD pathway [3]. None of the 28 other individuals of this first series showed allelic imbalance, according to our statistical criteria (Figure 4-B). The second series of analysis used SNP rs2236141 as marker and included 17 heterozygous individuals for this coding SNP. Eight of them were also heterozygous for SNP rs2236142 and had already tested negative for DAE with the first marker. The statistical threshold for DAE was not reached in any of the remaining 9 samples (Figure 3).

## Discussion

Our work supports the high sensitivity of HRM for the detection and quantification of DAE. We have shown that HRM is able to detect DAE associated with NMD in LCLs carrying a non-sense mutation in *CHEK2*. Although no DAE was observed in the patients who do not carry the 1100delC mutation, the series investigated here was limited, and we cannot rule out that *cis*-regulatory variants in *CHEK2 *may lead to DAE in a tissue specific manner [24]. However, this later hypothesis could not be tested since no breast tissue was available from these patients.

The approach used in our study relies on subjects who are heterozygous for a coding SNP and allows relative quantification of allelic transcripts. This methodology has major advantages over more conventional methods for investigating DAE based on the comparison of gene expression between individuals as discussed elsewhere [8,10,20]. Since they come from the same tissue sample and have therefore been subjected to the same environmental influences (such as genetic *trans*-acting factors and experimental exposures, including mRNA degradation) both alleles should be equally expressed in the absence of *cis*-acting sequence variation or epigenetic effects affecting the expression of the target mRNA. Thus, the strength of this approach is that each allele acts as an internal control for confounding factors, disclosing *cis*-variation effects without being confounded by any *trans*-variation effects.

Here, we report a complete solution for HRM analysis that can be used on both the HR-1™ (1 single capillary) and LightScanner^® ^(384-well plate format) instruments, with the format depending on the required throughput. Access to DAE assessment technology can be cost prohibitive for many laboratories. HRM provides a good alternative when compared to methodologies based for instance, on the use of capillary electrophoresis for single-base extension assays, such as SnapShot assays [11], allele-specific quantitative real-time PCR [12] and microarray platform [9]. Advantages offered by HRM analysis include its rapidity, cost-effectiveness and security due to its closed-tube nature. Though the HR-1™ is reported to provide a better accuracy [28], both instruments performed well to identify the 4 carriers of the CHEK2*1100delC variant showing DAE in the absence of puromycin treatment in our study. However, given the number of samples to test, analysis with the HR-1™ instrument ended up being much more time consuming (Table 1). The results obtained with the LightScanner^® ^instrument showed that this methodology can be applied in larger-scale studies, provided that LCL material is available, while maintaining high accuracy and remaining cost-effective. Indeed, the protocol is relatively inexpensive since it only requires standard PCR reagents and a small amount of fluorescent probe.

The script we developed using R computing software was made compatible with both instruments and greatly reduces the time of analysis. Once HRM data are acquired, the normalization of the curves, peak heights measurements, ratios calculations and statistical analysis are performed automatically within less than 15 minutes for a set of 96 samples when using the LightScanner^® ^instrument. The output consists in a summary table of the peak heights, relative allelic ratios, and the Student's t-test values, as well as a plot on which DAE carriers are highlighted. The script can also display other information on demand, such as melting curve profiles which can be displayed for each replicate or by average of 4 replicates for each individual (see examples in Figure 3 and 4).

In DAE analysis by HRM, the peak heights obtained from the melting curve reflect the relative abundance of each allele's transcript. The reproducibility and precision of the assay are reasonable as seen in the small standard deviations associated with the calculations. The accuracy of the method was illustrated by the consistency of the allelic expression estimates across multiple replicates assay within the same individual sample. Genomic DNA ratios varied within a very narrow range, showing the excellent reproducibility and precision of the assay on DNA derived from LCL. The intra-sample variation in replicate analysis was higher for mRNA ratios than for DNA ratios, possibly owing to RNA stability. In addition, at low copy numbers of mRNA, the stochastic distribution of the RNA templates may be a major source of variation and hence affect the accuracy of DAE analysis, by generating disagreeing replicate results for instance [29].

In a DAE study, the main optimization issue is the ability to select a subset of 2-3 marker SNPs so that as many individuals as possible are heterozygous for at least one of the markers. Subsets of individuals giving the most heterozygotes at 2 loci should be chosen in order to maximize redundancy, and to self-check for error reduction. Unfortunately, in the present study, no individual heterozygous for both SNPs showed evidence of DAE. Detection of DAE in a candidate gene may be indicative of the presence of a coding or regulatory variant altering expression of the gene product. However, DAE-based approaches can point out the presence of a regulatory causative variant only if the subjects are heterozygous for the causative variant (and of course for the coding SNP serving as marker). In some situations, the coding SNP used to distinguish both alleles may be itself responsible for the observed DAE, or it can be in linkage disequilibrium (LD) with it, *i.e*. on the same haplotype. In the case of no LD between the marker and the functional variant, it is still possible to map the variant, as previously reported by others [30,31].

## Conclusions

Allele-specific expression assays can be applied to identify genetic variants located in regions essential for gene expression regulation or splicing. Thus, identification of a list of genes for which DAE has been detected would yield a considerable reduction of the amount of work, by focusing discovery effort on the subset of genes that are most likely to harbour coding or regulatory variants that may alter gene expression. The approach reported here allows revealing the existence of regulatory variations without directly identifying or requiring prior knowledge of specific *cis*-regulatory SNPs. DAE assays can also highlight the existence of epigenetic factors controlling gene expression [32].

Analysis of the relative allelic ratios of marker SNPs circumvents the issue of confounding *trans*-acting factors. Any significant differences in these ratios support the existence of DAE and hence, *cis*-acting polymorphisms determining gene expression. The primary goal of this type of study is to identify sequence variants that are likely to alter gene expression and gene product function, and thereby influence susceptibility to breast cancer. However, to demonstrate that some of these variants actually show disease association, large-scale epidemiological studies are required and may ultimately lead towards the identification of causal genetic factors responsible for susceptibility to disease. In the context of such high-throughput studies, instead of LCLs, one can use blood samples, a more accessible tissue than breast. Identification and elucidation of rare intermediate-risk genetic variants associated with susceptibility to cancer will contribute to a better understanding of the aetiology of the disease.

## Competing interests

The authors declare that they have no competing interests.

## Authors' contributions

TN-D carried out the DAE experiments and drafted the manuscript; LPJ participated in the study design and helped to draft the manuscript; JM carried out the cell culture; NF and SM-C carried out the mutation screening; kConFab and OS provided the cell lines; FL-K participated in the development of the laboratory workflow and helped to draft the manuscript; MCS participated in the experiment design; SVT conceived the study, participated in its design and coordination and helped to draft the manuscript. FL participated in the study coordination and helped to draft the manuscript. All authors read and approved the final manuscript.

## Software availability

A copy of our R script code has been made available on http://Sourceforge.net.

Project name: HRMdae project;

Project home page: http://sourceforge.net/projects/hrmdae;

Operating system(s): Platform independent, R environment;

Programming language: R v2, or above;

Licence: GPL v3;

Any restrictions to use by non-academics: None.

## Pre-publication history

The pre-publication history for this paper can be accessed here:

http://www.biomedcentral.com/1755-8794/4/39/prepub

## Supplementary Material

Additional file 1 **Mutation screening results for the 41 breast cancer samples enrolled in the DAE study**. Additional table showing the mutation screening results for the 41 breast cancer samples enrolled in the DAE study. Click here for file

Additional file 2 **Primers and probes used in the DAE study on the *CHEK2 *gene**. Additional table showing the primers and probes sequences used to perform the DAE study. Click here for file
