# Supplementary material for: Detecting differential allelic expression using high-resolution melting curve analysis: application to the breast cancer susceptibility gene CHEK2
Source: BMC Med Genomics. 2011 May 11;4:39. doi: 10.1186/1755-8794-4-39 (PMC3112061; doi:10.1186/1755-8794-4-39)
Supplement: Additional file 1 — Mutation screening results for the 41 breast cancer samples enrolled in the DAE study. Additional table showing the mutation screening results for the 41 breast cancer samples enrolled in the DAE study. [file 1755-8794-4-39-S1.PDF]

Additional file 1 - Mutation screening results for the 41 breast cancer samples enrolled in the DAE study

Sample ID	Genotype at rs2236142[a]	Genotype at rs2236141[b]	1100delC carrier	Other variants (HGVS nomenclature)[c]	DAE results[d]	
					DAE value [95%CI]	p-value
1507	Het	CC		c.319+38_319+39insA	-0.16 [-0.22; -0.10]	0.0027
1526	Het	Het			-0.10 [-0.26; 0.07]	0.16
1802	CC	Het			-0.01 [-0.06; 0.04]	0.59
1928	Het	CC			-0.02 [-0.27; 0.23]	0.80
1967	Het	CC		c.319+38_319+39insA	0.01 [-0,06; 0.08]	0.47
2026	Het	Het			-0.14 [-0.29; 0.00]	0.049
2166	Het	CC		c.319+38_319+39insA	0.02 [-0.09; 0.14]	0.60
2181	Het	CC	Yes	c.319+38_319+39insA	-0.55 [-0.58; -0.48]	1.10-4
2193	Het	Het		c.252A>G	-0.07 [-0.25; 0.11]	0.30
2212	Het	CC		c.252A>G+c.319+38_319+39insA	-0.01 [-0.51; 0.49]	0.94
2234	GG	Het			-0.11 [-0.14; -0.09]	4.10-5
2247	Het	CC		c.319+38_319+39insA	0.01 [-0.33; 0.36]	0.90
2443	Het	CC			-0.03 [-0.07; 0.01]	0.095
2472	Het	CC		c.319+38_319+39insA	-0.01 [-0.08; 0.05]	0.62
2497	GG	Het			-0.02 [-0.05; 0.01]	0.16
2498	Het	CC	Yes	c.319+38_319+39insA	-0.37 [-0.53; -0.20]	0.0054
2499	GG	Het			-0.01 [-0.09; 0.07]	0.77
2500	Het	CC	Yes		-0.54 [-0.66; -0.25]	0.039
2526	Het	CC			0.04 [-0.16; 0.24]	0.61
2529	Het	CC			-0.14 [-0.25; -0.04]	0.020
2534	CC	Het			0.01 [-0.06; 0.07]	0.058
2536	Het	CC			0.06 [-0.03; 0.15]	0.12
2539	Het	CC			-0.05 [-0.23; 0.13]	0.43
2541	Het	CC			-0.07 [-0.31; 0.17]	0.43
2542	Het	CC			0.08 [-0.36; 0.52]	0.60
2557	GG	Het			-0.13 [-0.16; -0.10]	5.10-4
2570	GG	Het			-0.01 [0.07; 0.06]	0.82
2574	GG	Het			0.01 [-0.03; 0.04]	0.70
2665	Het	CC			-0.23 [-1.14; 0.68]	0.48
2666	Het	CC	Yes	c.319+38_319+39insA	-0.60 [-0.89; -0.47]	0.037
2667	Het	CC			-0.15 [-1.33; 1.03]	0.71
2668	Het	Het		c.444+24C>T	-0.08 [-0.55; 0.39]	0.64
2669	Het	Het			-0.11 [-0.21; -0.01]	0.035
2670	Het	Het			-0.19 [-0.33; -0.09]	0.0092
2671	Het	Het			-0.17 [-0.41; 0.30]	0.16
2674	Het	CC			0.05 [-0.02; 0.12]	0.12
2677	Het	Het			-0.06 [-0.41; 0.30]	0.63
2678	Het	CC			-0.01 [-0.15; 0.13]	0.85
2679	Het	CC		c.319+38_319+39insA	0.00 [-0.04; 0.03]	0.84
2680	Het	Het			0.05 [-0.14; 0.03]	0.84
2691	GG	Het			0.01 [-0.05; 0.07]	0.58

[a] GG, frequent homozygote; Het, heterozygote; CC, rare homozygote.
[b] CC, frequent homozygote; Het, heterozygote.
[c] Number based on transcript sequence (NM_007194), +1 as A of ATG start codon.
[d] DAE is expressed as the difference between the log of the signal ratio of the cDNA from the patient's LCL and the corresponding log ratio of genomic DNA.
