# Supplementary material for: Detecting differential allelic expression using high-resolution melting curve analysis: application to the breast cancer susceptibility gene CHEK2
Source: BMC Med Genomics. 2011 May 11;4:39. doi: 10.1186/1755-8794-4-39 (PMC3112061; doi:10.1186/1755-8794-4-39)
Supplement: Additional file 2 — Primers and probes used in the DAE study on the CHEK2 gene. Additional table showing the primers and probes sequences used to perform the DAE study. [file 1755-8794-4-39-S2.DOC]

*Additional file 2****:*** *Primers and probes used in the DAE study of* CHEK2.

| Specific Primer^¥^ or Probe |  | Oligonucleotide sequence |
| --- | --- | --- |
| Primary PCR on genomic DNA | Forward primer | 5’-GCAAAGAGAGCGTCTAACCAG-3’ |
|  | Reverse primer | 5’-GCAGAGTGGCGCTAAACCT-3’ |
| Primary PCR on cDNA | Forward primer | 5’-ATCTAGCCGTGGTCACTCGT-3’ |
|  | Reverse primer | 5’-TAGGACCCACTTCCCTGAAA-3’ |
| Secondary PCR | Forward primer | 5’-CAAAGAGAGCGTCTAACCAGACTAAT-3’ |
|  | Reverse primer | 5’-CAGATACAAACTCCACCCTCAGC-3’ |
| Simpleprobe for rs2236142^*^ | | 5’-TAAGTTCCGCTCT**C**CCTTCTAAA-3’ |
| Simpleprobe for rs2236141^*^ | | 5’-TCCTCATTGGTCC**G**GCGG-3’ |

^*^Polymorphic position is indicated in bold.

^¥^Marker SNPs rs2236141 and rs2236142 were located in the same amplicon.
